# Hydrogen Production through Glycerol Photoreforming on TiO_2_/Mesoporous Carbon: Influence of the Synthetic Method

**DOI:** 10.3390/ma13173800

**Published:** 2020-08-28

**Authors:** Juan Carlos Escamilla, Jesús Hidalgo-Carrillo, Juan Martín-Gómez, Rafael C. Estévez-Toledano, Vicente Montes, Daniel Cosano, Francisco J. Urbano, Alberto Marinas

**Affiliations:** 1 Departamento de Química Orgánica, Instituto Universitario de Investigación en Química Fina y Nanoquímica (IUNAN), Edificio Marie Curie, Campus de Rabanales, Universidad de Córdoba, E-14071 Córdoba, Spain; qo2esmej@uco.es (J.C.E.); juanmartingomez@outlook.es (J.M.-G.); q72estor@uco.es (R.C.E.-T.); q92cohid@uco.es (D.C.); fj.urbano@uco.es (F.J.U.); 2 Department of Chemical Engineering and Physical Chemistry, Faculty of Science University Institute of Water, Climate Change and Sustainability (IACYS), University of Extremadura, 06006 Badajoz, Spain; vmontes@unex.es

**Keywords:** TiO_2_/carbon composites, sol-gel process, glycerol photoreforming, hydrogen production

## Abstract

This article explores the effect of the synthetic method of titanium dioxide (TiO_2_)/C composites (physical mixture and the water-assisted/unassisted sol-gel method) on their photocatalytic activity for hydrogen production through glycerol photoreforming. The article demonstrates that, apart from a high surface area of carbon and the previous activation of its surface to favor titania incorporation, the appropriate control of titania formation is crucial. In this sense, even though the amount of incorporated titania was limited by the saturation of carbon surface groups (in our case, ca. 10 wt.% TiO_2_), the sol-gel process without water addition seemed to be the best method, ensuring the formation of small homogeneously-distributed anatase crystals on mesoporous carbon. In this way, a ca. 110-fold increase in catalyst activity compared to Evonik P25 (expressed as hydrogen micromole per grams of titania) was achieved.

## 1. Introduction

The use of fossil fuels as the main source of energy since the Industrial Revolution has led to a progressive increase in CO_2_ emissions, largely responsible for global warming and the greenhouse effect. The increase in population and evolution of the world economy has also resulted in an increasing energy demand, while fossil fuels are being depleted [1,2,3]. Therefore, there is a need for a transition from a fossil fuel-based economy to one based on renewables, which would allow us to limit global warming to well below 2 °C, in line with the Paris Agreement on climate change [1,4]. Hydrogen has the potential to be one of the clean fuels of the future, since, compared to fossil fuels, it has a high energy yield per unit mass of 122 KJ/g (2.75 times higher than that of fossil fuels) [5] and the only by-product released after combustion is water. However, the most widespread hydrogen production methodology is hydrocarbon (mainly natural gas) steam reforming, with water electrolysis only accounting for 3.9% of total cases [5,6]. Therefore, in order to obtain green hydrogen, it should be produced from non-fossil fuels, with biomass representing an interesting option. In this sense, the photocatalytic reforming of oxygenated organic compounds has been presented as an attractive alternative for biohydrogen production [7,8,9].

The most widely used photocatalyst is titanium dioxide (TiO_2_) due to its availability, chemical stability, price, and resistance to photo-corrosion [10,11]. However, this semiconductor has two drawbacks: The rapid recombination of electron-hole pairs, with around 90% or more of the photo-generated electrons recombining within ca. 10 ns, and the small use of sunlight, due to its high band gap value (3.2 eV) [12]. Several approaches have been developed to overcome these challenges, such as (i) doping with metals [13], which can result in a shift of the light absorption to visible light, due to the electrons found in the d orbitals of metals, which would cause a “new band” below the conduction band, lowering the band gap (Eg) [14]. Metals could also serve as electron traps due to their redox potentials [15]; (ii) non-metal doping [16]; (iii) co-doping [17,18]; or (iv) coupling with other semiconductors [19,20], to cite just some examples. Another solution for dealing with these problems could be the combination of TiO_2_ with a carbonaceous support [21]. The benefit of this approach is associated with the characteristics of carbonaceous materials. Firstly, they increase the surface area of the catalyst, favoring the dispersion of titania. Additionally, due to the increased porosity, they allow concentration of the organic compound on the surface of the support, facilitating its decomposition by activated TiO_2_ [22,23,24]. Secondly, it has been reported that TiO_2_-carbon composites can absorb in the visible range thanks to the photosensitizing effect of carbon, which would reduce the band gap value of TiO_2_ [25,26,27]. Finally, carbon can also function as a trap for the photoinduced electrons of the electron-hole pair of TiO_2_, delaying or hindering the recombination. An additional point to take into account is the role of surface groups present in carbon during the photocatalytic process. Matos et al., working with different TiO_2_-activated carbon composites, evidenced the importance of oxygenated groups on the surface of active carbon in photoactivity, granting an electronic density to titanium dioxide [28]. Ocampo-Perez et al. have also suggested that carboxylic groups act as catalytic reaction centers, capturing the electrons and reducing the recombination rate of the electron/hole pairs [29].

The aim of the present piece of research is to study the influence of the method of TiO_2_ incorporation on mesoporous carbon on the hydrogen photocatalytic production through glycerol photoreforming. The solids will be characterized by a wide range of techniques, in order to cast further light on the most important parameter(s) leading to the improvement of titania activity.

## 2. Materials and Methods

### 2.1. Materials

TiO_2_ (Evonik, Essen, Germany, P25), titanium (IV) isopropoxide (Sigma-Aldrich, St. Louis, MO, USA, 97%), glycerol (Sigma-Aldrich, St. Louis, MO, USA, 99%), 2-propanol (Sigma-Aldrich, St. Louis, MO, USA, >99.8%), mesoporous carbon (Sigma-Aldrich, St. Louis, MO, USA, <500 nm particle size (DLS) >99.95% trace metals basis), HNO_3_ (Sigma-Aldrich, St. Louis, MO, USA, >65%), H_2_SO_4_ (Panreac, Castellar del Vallès, BCN, Spain, 95–98%), HCl (Panreac, Castellar del Vallès, BCN, Spain, 37%), NaOH (Panreac, Castellar del Vallès, BCN, Spain, ≥98%), Na_2_CO_3_ (Panreac, Castellar del Vallès, BCN, Spain, ≥99.5%), NaHCO_3_ (Panreac, Castellar del Vallès, BCN, Spain, ≥99%), and deionized water (resistivity ≥18 MΩ cm, MilliQ Millipore, Billerica, MA, USA) were used in this study.

### 2.2. Synthesis of the Photocatalysts

Different photocatalysts consisting of titania-based solids physically mixed or supported on carbon were synthesized. Commercial mesoporous active carbon, called MC, was used as is or functionalized by an acid treatment, as described below.

#### 2.2.1. Mesoporous Carbon Functionalization (MC-H)

From the commercial mesoporous carbon (MC), several carbonaceous supports functionalized by acid treatment were synthesized, thus leading to solids called MC-H. The functionalization consisted of treating MC in an oxidizing medium (H_2_SO_4_/HNO_3_, 3:1 v/v), for which 10 mL of the oxidizing mixture was poured onto 1.0 g of MC, keeping the mixture in an ultrasonic bath at 25 °C for three hours. Then, the obtained solid was washed with deionized water until reaching a pH of 1.5, 2.0, or 3.0, as required. The solids were filtered using a Polytetrafluorethylene (PTFE) membrane with a pore size of 0.4 µm and were subsequently dried at 110 °C for 6 h and sieved on a 150 µm mesh. For the synthesis of TiO_2_/MC-H composites, the solid was washed until a pH of 1.5 was obtained.

#### 2.2.2. Physical Mixture Synthetic Method (PM)

A photocatalyst was obtained by physically mixing Evonik P25 titanium dioxide and the non-functionalized carbonaceous support MC at a TiO_2_/MC ratio of 40% wt. For this, 0.4 g of P25 was mixed with 0.6 g of MC and 50 mL of deionized water was added. The mixture was kept under constant stirring (1100 rpm) for 1 h at 70 °C. After the homogenization time had elapsed, water was evaporated on a rotary evaporator, and the resulting solid was dried at 110 °C for 12 h and then calcined at 400 °C for 2 h, sieved on a 150 µm mesh, and stored until use. The solid obtained was named PM(40%)TiO_2_/MC.

#### 2.2.3. Ultrasound-Assisted Sol-Gel Synthetic Method (SG1)

A first series of photocatalysts based on the sol-gel (SG) method were prepared from a carbonaceous support (MC or MC-H) on which TiO_2_ was deposited from a titanium isopropoxide precursor that was hydrolyzed without water addition (SG1 variant). TiO_2_ was incorporated on the carbonaceous support at nominal loadings of 10, 25, and 40% wt. of TiO_2_, as required. Taking the example of the nominal loading of 40% wt. TiO_2_, the procedure was as follows: 1.0 g of MC (or MC-H) was added to a solution containing 2.5 mL of titanium tetraisopropoxide dissolved in 10 mL of isopropanol, introducing the mixture in an ultrasonic bath for 4 h at 25 °C to achieve perfect homogenization. Subsequently, the homogenized mixture was refluxed at 80 °C for 24 h, in order to achieve the hydrolysis of TiO_2_ on the surface of the carbonaceous support. The solid obtained was recovered by filtering at 60 °C to facilitate the passage of the solvent in the membrane and, subsequently, it was dried at 110 °C for 12 h and calcined at 400 °C for 2 h. The solid was finally sieved through a 150 µm mesh and stored until use. Therefore, the catalysts named SG1(X%)TiO_2_/MC and SG1(X%)TiO_2_/MC-H were obtained, where X% indicates the percentage by nominal weight of TiO_2_ (10, 25 or 40 wt.%) that it is intended to be incorporated into the carbonaceous support.

#### 2.2.4. Water-Assisted Sol-Gel Synthetic Method (SG2)

In order to facilitate the incorporation of TiO_2_ on the carbonaceous support, a modification of the sol-gel method was carried out, in which the hydrolysis of titanium isopropoxide was achieved by adding water to the reaction medium (SG2 series). Therefore, a solid with a weight ratio of 40 wt.% TiO_2_/MC was prepared, starting from a solution containing 2.5 mL of titanium tetraisopropoxide dissolved in 10 mL of isopropanol, to which 1.0 g of MC was added. The mixture was homogenized for 30 min and, subsequently, 20 mL of deionized water was added dropwise, until the sol was formed. Aging was carried out under constant stirring (800 rpm) for 12 h at a temperature of 25 °C. The solid obtained was filtered, dried, calcined, and sieved under the same conditions as those employed for the SG1 series. Therefore, the solid named SG2(40%)TiO_2_/MC was obtained.

### 2.3. Catalyst Characterization

The determination of acid functional groups of MC and MC-H was carried out by the Boehm method [30,31]. The method consists of a selective neutralization of surface groups, such as carboxyl, lactonic, and phenolic groups with bases of different strengths. Total surface acid groups were determined by mixing 20 mg of mesoporous carbon with 25 mL of a 0.01 M NaOH solution, which was kept under constant stirring at 100 rpm for 5 days at 25 °C. The supernatant was then filtered on a 0.4 µm pore size PTFE membrane syringe filter and a 10 mL aliquot was taken and titrated with a 0.01 M HCl solution. The change in pH was recorded with a pH meter (Ag/AgCl electrode and EC-Meter GLP 31 equipment). The number of total acid functional groups is a function of the volume of HCl consumed until neutrality. The quantification corresponding to carboxylic acids plus lactones was carried out by following the previous procedure, but using a 0.01 M Na_2_CO_3_ solution as the reaction medium. For the carboxylic acids, a 0.01 M NaHCO_3_ solution was utilized and phenols were calculated by difference.

The point of zero charge (PZC) of the solids was determined by the mass titration method by suspending variable amounts of MC or MC-H (20–600 mg) in 10 mL of a 0.1 M NaCl solution, using deionized water as the solvent. Suspensions were stirred for 12 h before pH measurements with a pH-meter [32,33].

Chemical analyses were carried out by X-ray fluorescence on a Rigaku ZSX Primus IV spectrometer (Rigaku, Austin, TX, USA), equipped with a 4 kW rhodium anode, a proportional gas flow detector for light elements, and a scintillation counter for heavy elements.

X-ray photoelectron spectroscopy (XPS) data were recorded at the Central Service for Research Support (SCAI) of the University of Córdoba (Córdoba, Spain) on pellets (4 mm diameter, 0.5 mm thick) after outgassing the samples to a pressure below 2 × 10^−8^ Torr at 150 °C. A Leibold–Heraeus LHS10 spectrometer (SPECS, Berlin, Germany) was operated with an AlKα X-ray source (hν = 1486.6 eV) at 120 W and 30 mA, using C (1s) as the energy reference (284.6 eV).

Textural properties were determined through N_2_ adsorption-desorption isotherms at the boiling temperature on Micromeritics ASAP 2010 equipment (Micromeritics, Norcross, GA, USA). Before measurements, all samples were outgassed at 0.1 Pa at 120 °C.

Field-emission scanning electron microscopy (FESEM) images were acquired with a Jeol JSM-7800F Prime microscope (JEOL Ltd., Tokyo, Japan) available at the microscopy unit of the Central Service for Research Support (SCAI) facility of the University of Córdoba (Córdoba, Spain). This equipment is furnished with an EDX analyzer, allowing us to record the mapping of elements.

X-ray diffraction (XRD) patterns were recorded in the 5–80° range on a Bruker D8 Discover A25 diffractometer (Bruker Española S.A., Madrid, Spain) equipped with Cu Kα radiation, a Ge monochromator, and a Lynxeye detector.

Raman spectra were obtained with InVia Raman equipment (Renishaw Ibérica, S.A.U., Barcelona, Spain) furnished with a Leica microscope and a Charge Coupled Device (CCD) detector. Spectra were collected with a green laser (532 nm) in the 100–1800 cm^−1^ Raman shift range, accumulating 20 spectra.

IR spectra were obtained on a FT-MIR Bruker Tensor 27 spectrometer at the NIR/MIR unit of the Central Service for Research Support (SCAI) of the University of Córdoba (Córdoba, Spain).

Diffuse reflectance UV-Vis spectroscopy was performed on a Cary 1E (Agilent, Santa Clara, CA, USA) instrument, using BaSO_4_ as reference material.

### 2.4. Photocatalytic Hydrogen Production

Glycerol photoreforming was carried out in a 190 cm^3^ cylindrical reactor with a catalyst concentration of 0.5 g/L in a glycerol solution (10% v/v) that was kept under constant stirring. The reaction was carried out by means of an ultraviolet irradiation source produced by a mercury lamp (125 W, Photochemical Reactors Ltd., Reading, UK) for 6 h. The irradiation range of the lamp used is 254–579 nm, with a maximum at 365–366 nm. The gases produced in the reaction were swept by a flow of Ar (20 mL/min) towards a Hyden HR20 mass spectrometer (Hyden Analytical Ltd., Warrington, UK), where H_2_ and CO_2_ were continuously monitored (m/z values of 2 and 44, respectively). The lamp power at the sample compartment, as measured at <800 nm with Ophir Starlite equipment, was 116 mW·cm^−2^. 

## 3. Results

### 3.1. Functionalization of MC to Obtain MC-H Solid

The functionalization of carbon leads to the creation of surface functional groups, such as carboxylic acids, lactones, and/or phenols, which reduces the hydrophobicity of the catalyst surface. This could favor subsequent incorporation of the semiconductor (TiO_2_) and improve the adsorption-desorption of glycerol or reaction intermediates during the photocatalytic reaction in aqueous medium [34,35]. In our case, commercial mesoporous carbon (MC) was functionalized by treatment with a mixture of H_2_SO_4_:HNO_3_ (3:1 v/v) to obtain the solid MC-H. At the end of the activation process, the solid was washed with deionized water until obtaining a pH in the washing water of 1.5, 2.0, or 3.0, with the idea of achieving carbon with different degrees of functionalization.

Table 1 shows the different acidic groups obtained by Boehm titrations, as well as the pH at the point of zero charge (PZC) for the diverse carbon. The results indicate that non-functionalized MC has a PZC value close to neutrality, while the values obtained for MC-H decrease in parallel with the pH at which the functionalized solids are washed. Therefore, the MC-H solids washed at pH values of 1.5, 2.0, and 3.0 show a PZC of ca. 2.8, 3.5, and 3.7, respectively. In parallel to the decrease in the pH at the PZC, a progressive increase in total surface acidity can be observed, ranging from ca. 13.4 mmol·g^−1^ for MC to 17.8 mmol·g^−1^ for MC-H (pH = 1.5).

Regarding the distribution of superficial acidic functional groups, acid treatment results in an increase in the percentage of carboxylic acids to the detriment of weaker phenolic groups, whereas the distribution of lactonic ones is quite stable (23.3–24.7%). The observed increase in strong acid sites is consistent with data reported in the literature [36,37]. Sulfonic groups could also contribute to acid sites identified as carboxylic groups, since both are strong acid sites which cannot be distinguished by Boehm’s method [36].

With a view to the subsequent incorporation of TiO_2_ on the surface of the carbon, MC-H washed at pH 1.5 was chosen as the starting solid due to its greater total surface acidity, and the groups were expected to favor the process.

Taking the non-functionalized (MC) and functionalized (MC-H, pH = 1.5) carbonaceous materials and the corresponding titanium precursors as starting materials, a series of titania-mesoporous carbon composites were synthesized using two methods of synthesis. The first method consisted of a physical mixing procedure suspending TiO_2_ (Evonik P25) and the corresponding carbon in deionized water and, after stirring, removing the solvent, drying, and calcining, which led to solids with the prefix PM. As a second synthetic method, the sol-gel procedure was used, in which the carbon was suspended in a solution of titanium isopropoxide, inducing the formation of TiO_2_ by hydrolysis of the titanium isopropoxide on the surface of the carbon by refluxing under anhydrous conditions (SG1) or after adding a small amount of water (SG2). The nominal amount of TiO_2_ incorporated in the carbon was between 10 and 40% wt. The semiconductors thus synthesized were characterized from a chemical, textural, and structural point of view.

### 3.2. Characterization of TiO_2_/MC Catalysts

#### 3.2.1. Chemical Composition

The chemical composition of the different synthesized photocatalysts was investigated using X-ray Fluorescence (XRF). The results of the compositional analysis are shown in Table 2, where the weight percentage of TiO_2_ incorporated on the carbons and the sulfur content in the catalysts are presented. As can be seen, the efficiency of the incorporation of TiO_2_ into the different carbonaceous materials depends on the synthesis method. Therefore, the solids obtained by the physical mixing procedure (PM) and water-induced sol-gel (SG2) show the greatest efficiency in the incorporation, approaching a nominal content. On the other hand, for the sol-gel procedure assisted by sonication and in the absence of water (SG1), the efficiency was lower, and considerable differences between the actual and nominal content of TiO_2_ could be observed. In the most extreme case, for solid SG1(40%)TiO_2_/MC-H, it was only possible to incorporate approximately a quarter of the desired TiO_2_ content (10.5% of TiO_2_ obtained versus 40% nominal). Furthermore, within the SG1 procedure, the efficiency of TiO_2_ incorporation was better (closer to the nominal value) when the amount of titania to be incorporated was lower, which suggests that titania incorporation is limited by saturation of the carbon surface. In contrast, the hydrolysis of titanium isopropoxide induced by water in the SG2 method facilitates the formation of TiO_2_, resulting in almost 100% incorporation of the added precursor, although it is possible that such hydrolysis takes place partially outside the surface of the carbon and that isolated (not interacting with carbon) TiO_2_ particles could be obtained. Finally, in the PM solid, since it consists of a physical mixture of both solids already formed, the amount of TiO_2_ in the final solid is 100% of that desired. Taking all of the above into account, there are binary semiconductors with a TiO_2_ content ranging from ca. 6.6% in SG1(10%)TiO_2_/MC-H to 43.6% by weight in SG2(40%)TiO_2_/MC.

As for the sulfur content of the solids, the non-functionalized mesoporous carbon (MC) shows sulfur heteroatoms in its composition (ca. 1% S). The functionalization of carbon in H_2_SO_4_/HNO_3_ medium only led to a slight increase in the percentage content of sulfur in the solid MC-H (1.2% S), which could imply that the presence of superficial sulfonic groups from the acid treatment is limited. On the other hand, the method of the incorporation of TiO_2_ into the carbonaceous materials (either MC or MC-H) usually leads to a decrease in the sulfur content, probably as a consequence of possible leaching during the reflux treatment and/or the incorporation of a certain amount of TiO_2_ that is free of sulfur. In fact, the greatest loss of sulfur is observed in the semiconductor with the highest TiO_2_ loading and obtained by water-induced hydrolysis, as in the solid SG2(40%)TiO_2_/MC. This effect is clearer in photocatalysts synthesized from MC, whereas when MC-H is used as a carbonaceous support, the loss of S is less evident, which could suggest that there are different species of sulfur in both solids.

In order to cast further light on the nature of superficial sulfur and the modification of such species and carbon groups with the acid treatment of carbonaceous supports, additional XPS experiments were performed. Table 3 allowed us to compare SG1(40%)TiO_2_/MC and SG1(40%)TiO_2_/MC-H. In terms of the total atomic content, it is evident that the acid treatment resulted in an increase in the oxygen content (from 27.88% to 39.22%) at the expense of carbon (which decreased from 60.70 to 48.45%), whereas the sulfur content, in line with the XRF data shown in Table 2, was doubled (from 0.57% to 1.32%). Focusing on the nature of carbon species, activation of carbon with H_2_SO_4_/HNO_3_ resulted in an increase in the relative percentage of C1s peaks centered at 286.6–286.9 eV (associated with phenolic and alcohol groups), 287.7–288.0 eV (carbonyl or quinone groups), and 288.7–289.1 eV (ester and carboxylic groups) [38], to the detriment of polyaromatic, aromatic structures and satellite peaks due to π-π* transitions in aromatic rings (denoted as C (sp^2^), C (sp^3^), and π-π*, respectively). This is consistent with data obtained through Boehm titration, which evidenced the increase in surface acidic groups (i.e., the oxidation of surface groups) with the acid treatment. This is also confirmed by the increase in the relative intensity of O1s signals centered at ca. 532.1 and 533.4 eV (associated with alcohols and carboxylic groups, among others) [38,39]. The Ti2p3/2 signal at 459.0 eV is consistent with TiO_2_ [40].

As for sulfur-containing species, it is evident that acid treatment also results in an increase in the S2p3/2 signal centered at ca. 169.4–169.5 eV [41], associated with sulfate groups (relative percentages of 56.3 and 93.2% for SG1(40%)TiO_2_/MC and SG1(40%)TiO_2_/MC-H, respectively), to the detriment of the signal at ca. 164.1–164.6 eV (which is assigned in the literature to S_8_, aromatic sulfide groups, or C–S) [41,42,43].

#### 3.2.2. N_2_ Adsorption/Desorption Isotherms

The adsorption/desorption isotherms of N_2_ at its boiling temperature obtained for all of the catalysts were type IV isotherms, according to the IUPAC classification, corresponding to mesoporous materials. Table 2 shows the specific surfaces obtained for the synthesized solids. In general, it can be observed that the acid treatment of MC practically does not alter the specific surface of the resulting MC-H solid (220 m^2^·g^−1^). However, the subsequent incorporation of TiO_2_ into the composite clearly reduces the surface area of the solid obtained, regardless of the synthesis method or the amount of TiO_2_ incorporated. In the most drastic case of the SG1(10%)TiO_2_/MC-H catalyst, the surface area obtained was 147 m^2^·g^−1^, which implies a 33% reduction in the starting MC-H. This could indicate partial clogging of the porous structure with titania incorporation [26,44].

Table 4 includes the average pore diameter and pore volume data, as well as the distribution between micropores and mesopores of the catalysts. The results indicate that the acid treatment of MC led to an increase in the average pore diameter and pore volume (comparing MC-H and MC). On the other hand, the incorporation of TiO_2_ into carbonaceous materials resulted in a decrease in the volume of mesopores and thus the total volume, in line with the above-mentioned decrease in surface area.

#### 3.2.3. SEM Micrographs

**Figure 1:**
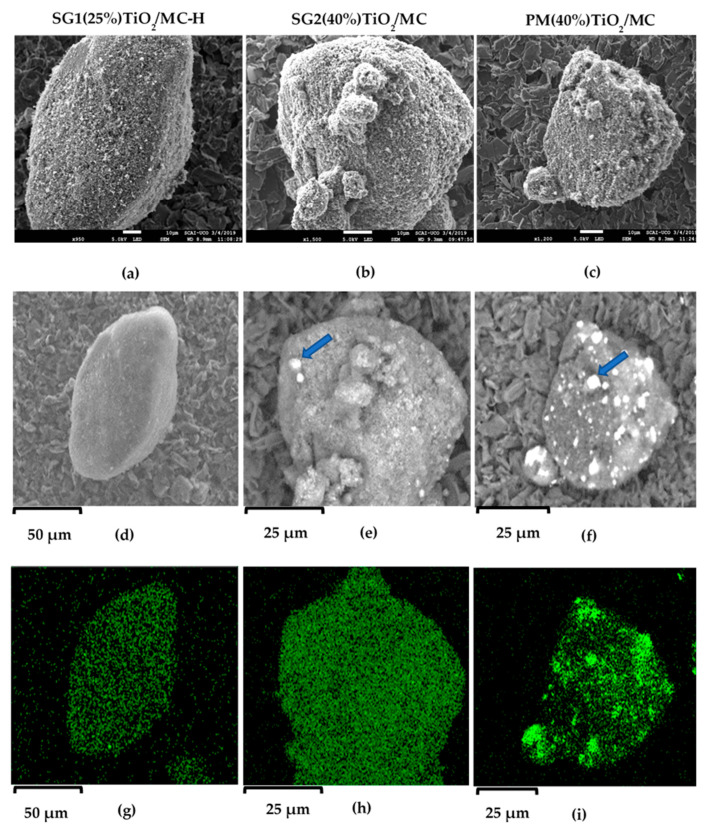
Secondary electron detector (SEM), backscattered electron (BSE), and Energy-dispersive X-ray spectroscopy (EDS) micrographs for three solids representative of each synthetic method: SG1(25%)TiO_2_/MC-H, SG2(40%)TiO_2_/MC, and PM(40%)TiO_2_/MC. (**a**) 5 kV × 950; (**b**) 5 kV × 1500; (**c**) 5 kV × 1200; BSE (**d**–**f**); EDS mapping obtained for the Ti Kα1 signal (**g**–**i**).

Figure 1 shows the micrographs corresponding to one representative catalyst of each synthetic method. The images obtained with the secondary electron detector (SEM) exhibit irregular particles with a rough surface in all of the photocatalysts, regardless of the synthetic method. However, it is important to highlight that SG1(25%)TiO_2_/MC-H (Figure 1a) displays a greater surface homogeneity than SG2(40%)TiO_2_/MC (Figure 1b) and PM(40%)TiO_2_/MC (Figure 1c), probably due to the ultrasonic treatment used during the synthesis and the fact that the hydrolysis process of titanium isopropoxide took place under milder (anhydrous) conditions.

Furthermore, the micrographs obtained with backscattered electrons (BSE), sensitive to the chemical composition of the samples, show that the incorporation of TiO_2_ into the carbonaceous material is much more homogeneous in the solid SG1(25%)TiO_2_/MC-H (Figure 1d) than in SG2(40%)TiO_2_/MC (Figure 1e), especially the PM(40%)TiO_2_/MC (Figure 1f), for which large TiO_2_ particles (marked with a blue arrow) can be clearly observed on the surface of the carbon particle. It must be remembered that in this latter synthetic method, the TiO_2_ incorporated is already formed, by means of a physical mixture with the carbonaceous support. The SG2(40%)TiO_2_/MC catalyst also exhibits large TiO_2_ particles (arrow in Figure 1e), probably as a result of the addition of water to the synthesis medium to favor the hydrolysis of titanium isopropoxide, which surely led to the formation of TiO_2_ particles independently of the carbonaceous support. The EDS (Ti, Kα1) mapping corroborates the previous observations; that is, a great surface homogeneity in SG1(25%)TiO_2_/MC-H (Figure 1g) and SG2(40%)TiO_2_/MC (Figure 1h), with the latter having a higher content of Ti (higher green intensity). The heterogeneity of the PM(40%)TiO_2_/MC solid is also evident in Figure 1i.

#### 3.2.4. X-ray Diffraction

**Figure 2:**
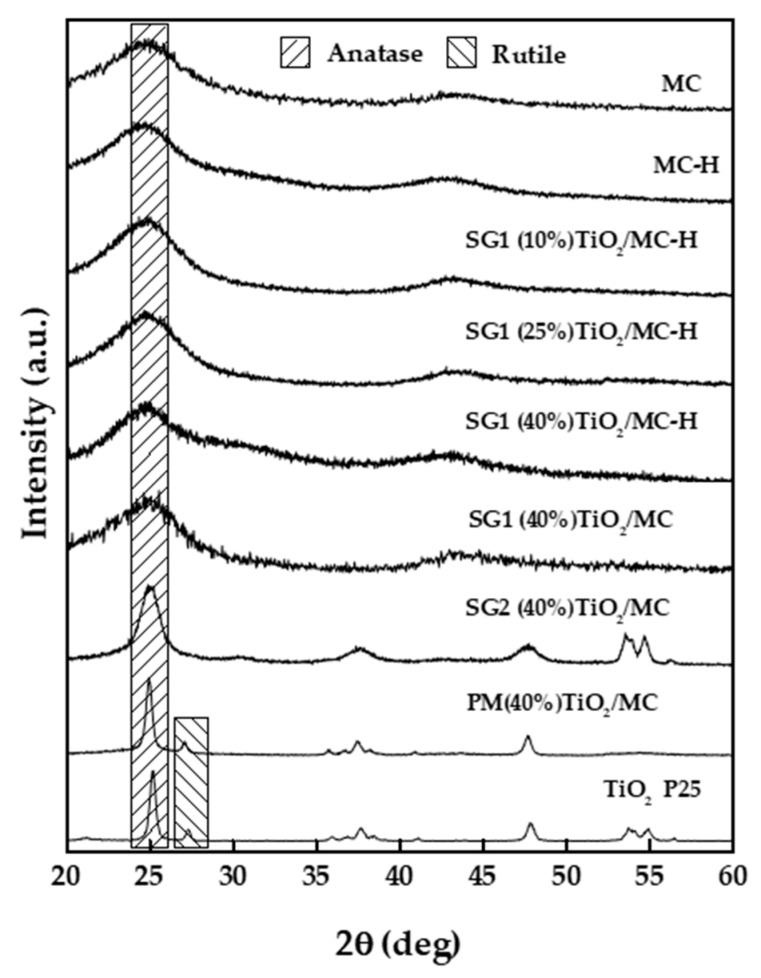
X-ray diffractograms of the different solids used in the present study.

Figure 2 depicts the X-ray diffractograms of the different catalysts used in this work. The Evonik P25 titanium dioxide diffractogram exhibits the anatase and rutile diffraction bands corresponding to its composition (ca. 80% anatase and 20% rutile). Therefore, peaks at 2θ values of 25.33°, 37.84°, 48.09°, 54.00°, and 55.13° correspond to (101), (004), (200), (105), and (211) lattice planes in anatase, respectively (JCPDS card Nr. 21-1272), whereas those at 27.48° and 36.09° are respectively associated with (110) and (101) lattice planes in rutile (JCPDS Card Nr. 21-1276) [45,46]. As for the commercial mesoporous carbon (MC), the broad and relatively low-intensity reflections at 23° (002) and 43° (100) associated with the degree of graphitization of the carbon indicate the disordered nature of the carbonaceous material [47,48], which is not significantly affected by the acid treatment. Raman spectroscopy is a more sensitive technique that can provide additional information in this regard. The catalyst synthesized by the physical mixture PM(40%)TiO_2_/MC also shows diffraction bands characteristic of anatase and, to a lesser extent, rutile, from the starting P25 TiO_2_. The catalyst prepared by the water-induced hydrolysis of titanium isopropoxide—SG2(40%)TiO_2_/MC—presents wide diffraction bands associated with the anatase phase of TiO_2_. Finally, in the solids prepared by the SG1 method, with the hydrolysis of the titanium isopropoxide in an anhydrous medium and therefore a smoother process, no clear bands associated with TiO_2_ are seen. This observation, like the previous ones, is in perfect agreement with the data obtained by SEM-BSE-EDX, which indicate that, by means of the SG1 method, TiO_2_ is deposited as small particles (crystallites) distributed very homogeneously on the surface of the carbonaceous material. In the case of the SG2 method, while also leading to a homogeneous distribution of TiO_2_ particles, forced hydrolysis of the titanium precursor led to larger TiO_2_ particles of SG2(40%)TiO_2_/MC, as seen in SEM micrographs and incipiently in the catalyst X-ray diffractogram.

#### 3.2.5. Raman Spectroscopy

**Figure 3:**
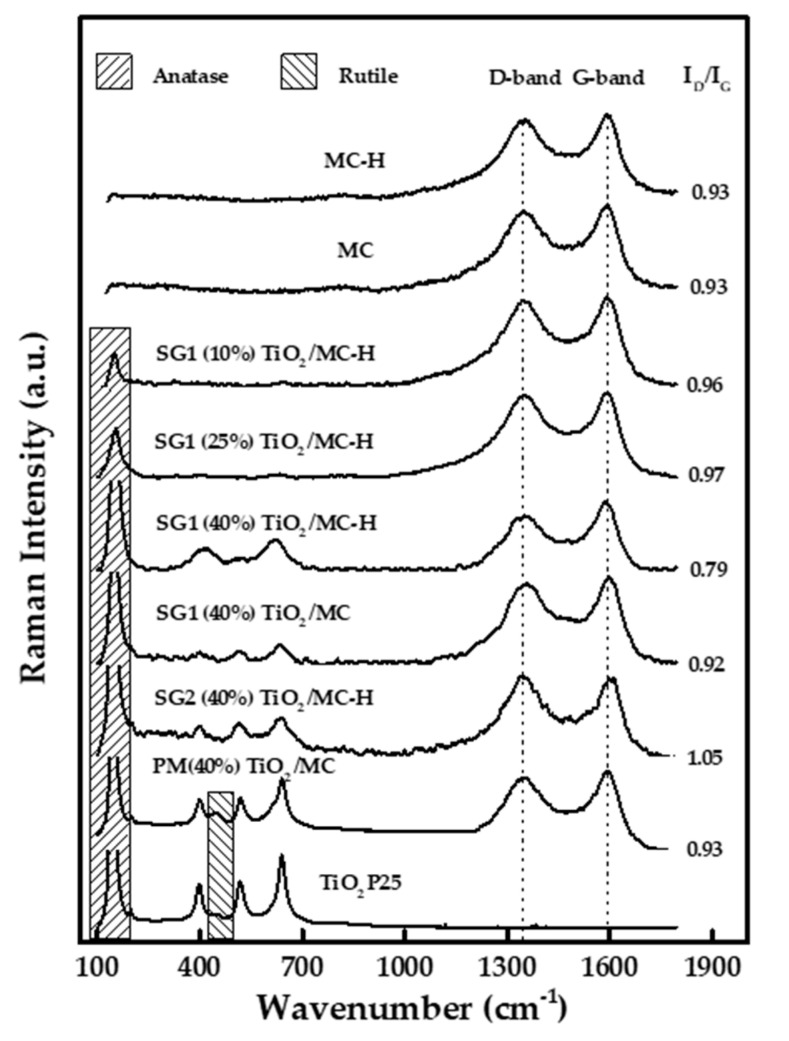
Raman spectra of some of the solids used in the present study.

Raman spectroscopy is a much more sensitive technique than X-ray diffraction and is employed to determine the structures of solids, such as titanium oxide. Figure 3 represents the Raman spectra of the different synthesized photocatalysts, as well as the spectra of Evonik P25. For this last solid, signals corresponding to anatase (major phase) are observed, with the vibration modes Eg (147 and 640 cm^−1^), B1g (398 cm^−1^), and A1g-B1g (515 cm^−1^), mainly, and rutile (minor phase), with the modes Eg (448 cm^−1^) and A1g (612 cm^−1^) [49,50]. The results obtained by Raman spectroscopy for the TiO_2_-carbon composites showed, in all cases, that TiO_2_ is mainly in the anatase phase (signals at 147 and 640 cm^−1^), although, in agreement with the results obtained by XRD, the signals observed in the solids synthesized by the sol-gel procedures (SG1 and SG2) are weaker and wider, which is associated with TiO_2_ nanoparticles having a small size and being homogeneously dispersed on the surface of the carbonaceous matrix. On the other hand, as expected due to the synthesis method, the PM(40%)TiO_2_/MC catalyst exhibits peaks corresponding to both anatase and rutile phases.

Raman spectroscopy has also been used in the last few decades to structurally characterize carbonaceous materials [51,52]. As can be seen in Figure 3, carbon-based solids exhibit two peaks centered at ca. 1595 and 1350 cm^−1^, denoted as the G and D peak, respectively. The former is a characteristic scattering peak of graphite, whereas the latter is associated with lattice defects, the disordered arrangement, and the low symmetry carbon structure of graphite. Therefore, the relative intensity of both signals is related to the structure order. The higher the intensity ratio of the D band to the G band (I_D_/I_G_ ratio), the higher the disorder in the structure [53,54]. The I_D_/I_G_ ratios of the photocatalysts are between 0.79 and 1.05, which denotes a high degree of disorder of the graphite sheet arrays. Moreover, it can be concluded that AC functionalization and TiO_2_ deposition do not significantly affect that structure [51]. In any case, the highest degree of order seems to have been obtained for SG1(40%)TiO_2_/MC-H.

#### 3.2.6. FT-IR Spectroscopy

**Figure 4:**
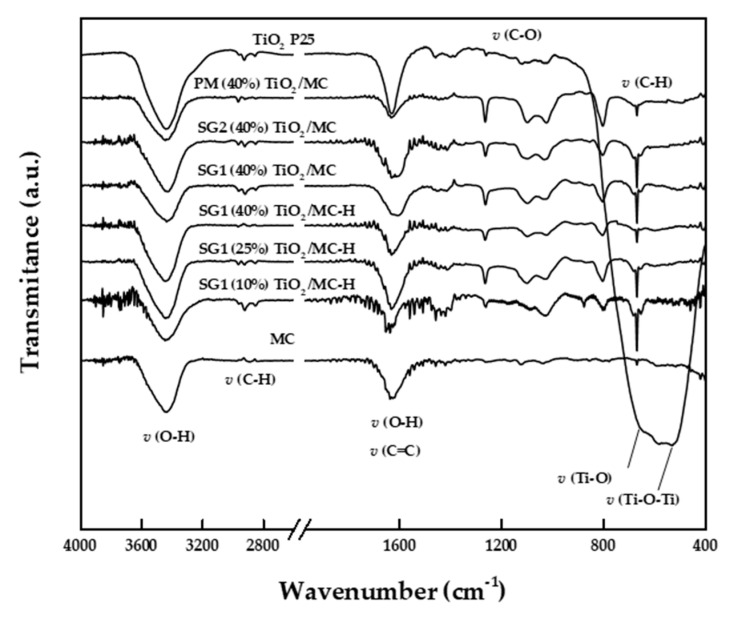
FT-IR spectra of the different catalyst in the 400–4000 cm^−1^ range.

Figure 4 shows the DRIFT spectra of the AC and Evonik P25 solids, as well as those of the different synthesized binary catalysts. Regarding P25, the spectrum basically exhibits a wide signal above 3200 cm^−1^ (centered at 3454 cm^−1^) and another at 1633 cm^−1^, corresponding, respectively, to the stretching and bending modes of the OH groups [55]. A wide and very intense signal at 650–800 cm^−1^ can also be observed, with maxima at 670 and 512 cm^−1^ associated with the Ti–O and Ti–O–Ti stretching vibrations, respectively [56,57]. The spectrum also shows bands centered at 2922 and 2855 cm^−1^ assigned to C–H stretching, probably due to organic residues. Similar results have been described in the literature for P25 samples [58]. As for the carbonaceous material MC, it has an OH bond stretching band above 3200 cm^−1^ (those of C–H stretching in alkanes are a bit below 3000 cm^−1^), as well as signals at 1629 cm^−1^ (associated in the literature with OH bending and/or C=C stretching) [39,56] and 1000–1300 cm^−1^ (C–O stretching) [59,60]. Regarding binary catalysts, they exhibit a profile more similar to that of MC, with OH stretching centered at 3430–3440 cm^−1^; C–H stretching bands at ca. 2960 and 2940 cm^−1^; OH bending and/or C=C stretching at ca. 1630; and several bands at 1100–1300 cm^−1^, which are assigned in the literature to C–O stretching modes in different oxygenated groups (ca. alcohol, phenol, and carboxylic acids) [52,60]. Finally, in the 550–800 cm^−1^ region, there are also several bands which could be due to both Ti–O vibrations and C–H aromatic bending [56,61].

In the case of MC-H series, FT-IR bands do not allow us to clearly distinguish possible bands of nitrogen (e.g., nitro groups at ca. 1330–1530 or 1574 cm^−1^) [62] or sulfur-containing species (e.g., sulfate groups at 1000–1250 cm^−1^) [63] resulting from surface functionalization, probably due to their small content and overlap with some other bands. In any case, the above-mentioned XPS results evidenced the presence of sulfur species in both MC and MC-H series, which were more oxygenated in the latter case.

#### 3.2.7. UV-Vis Spectroscopy

**Figure 5:**
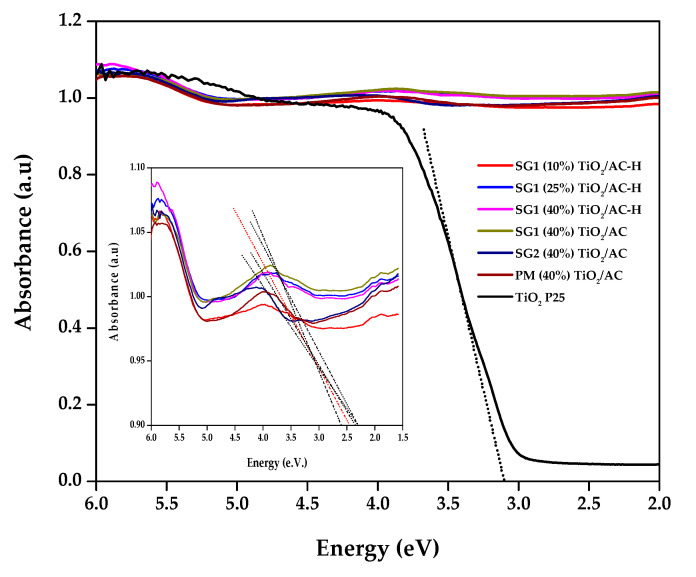
Diffuse reflectance UV-Vis spectra of the different catalysts used in the present study.

Figure 5 represents the diffuse reflectance UV-Vis spectra of the synthesized photocatalysts, while Table 2 shows the band gap values (Eg) calculated by the Tauc method (Figure S1) [64,65]. The value obtained for P25 was 3.15 eV, thus only absorbing radiation at wavelengths below 400 nm. On the other hand, the Eg values obtained for the catalysts that include carbonaceous materials are in the 2.25–2.37 eV range and their spectra show continuous absorption throughout the recorded spectrum. This is consistent with the data reported for other TiO_2_–C composites [54], evidencing absorption in the visible spectrum. Finally, there is no clear relationship between the Eg values obtained and the synthesis method used (SG1, SG2, or PM).

#### 3.2.8. Glycerol Photoreforming

The overall reaction for glycerol photoreforming is

(1) $$C_{3}H_{8}O_{3}+3H_{2}O\to 3{\mathrm{CO}}_{2}+7H_{2}$$

For a detailed mechanistic study, see [66].

**Figure 6:**
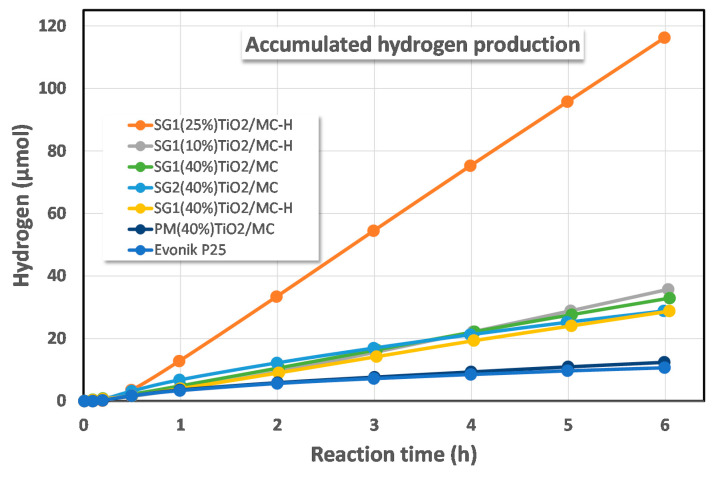
Accumulated hydrogen production from aqueous glycerol photoreforming for the synthesized TiO_2_-MC binary composites.

All synthesized catalysts were tested in the H_2_ production reaction by glycerol photoreforming in the aqueous phase. Figure 6 shows the accumulated H_2_ production profiles obtained for each of the solids. The catalysts tested can be classified into three groups, depending on the activity shown in the process. Therefore, a first group of small active catalysts is formed by the Evonik P25 catalyst, together with the solid PM(40%)TiO_2_/MC obtained by the physical mixture of P25 and MC in suspension. However, it should be noted that the activity of this last catalyst is slightly higher than that of P25, bearing in mind that, when using the same weight of catalyst, the net amount of TiO_2_ in this mixture is only 40% of the total. These are reasonable results that prove that the physical mixing procedure does not produce a substantial improvement in catalytic activity or that, in other words, more intimate contact between TiO_2_ and activated carbon would be necessary for an improvement to be observed. In a second group of catalysts are the solids SG1(40%)TiO_2_/MC-H, SG2(40%)TiO_2_/MC, SG1(40%)TiO_2_/MC, and SG1(10%)TiO_2_/MC-H, with an activity clearly superior to that of the previous catalysts. In this group, there are solids synthesized by SG1 and SG2 methods and on MC and MC-H as supports. There are also catalysts that have an actual TiO_2_ loading that ranges from just 6.6% in SG1(10%)TiO_2_/MC-H to 43% in SG2(40%)TiO_2_/MC. Finally, SG1(25%)TiO_2_/MC-H is found as the most active catalyst, which generates three times more hydrogen than catalysts included in the second group of photocatalysts. It is a solid synthesized by the SG1 procedure, the differential features of which are the performance of hydrolysis of the titania precursor by refluxing at 80 °C and the absence of water in the synthesis medium. As previously mentioned, it is a catalyst that, despite having an actual TiO_2_ loading of only 10% by weight, is optimally dispersed on the surface of the MC-H, as evidenced by the SEM-BSE-EDX images of Figure 1. In addition, this catalyst was tested in a reaction for more than 20 h, showing no signs of deactivation, increasing the amount of hydrogen generated in an almost linear manner (Figure S2).

The improvement observed for TiO_2_-carbon composites compared to pure TiO_2_ is usually attributed to an increase in the surface area and retardation of the electron-hole recombination [67].

**Figure 7:**
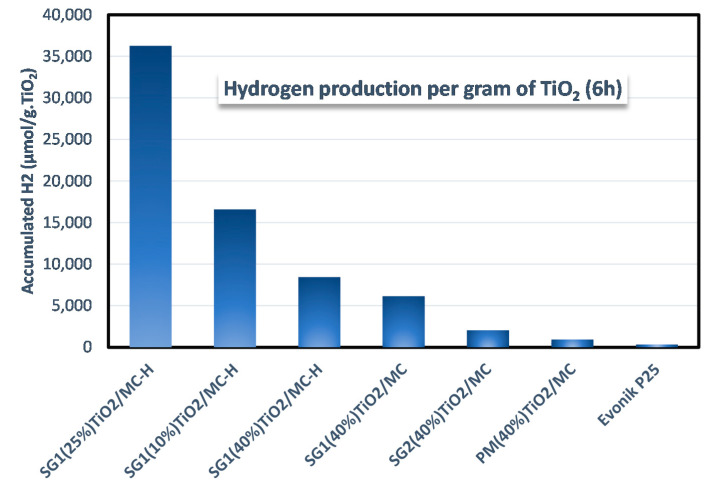
Accumulated hydrogen production after 6 h of reaction expressed as µmol of H_2_ per gram of TiO_2_ for all the photocatalysts used in this work.

As commented by Leary and Westwood [67], there are studies in the literature that compare TiO_2_-carbon composite photocatalysts to a pure TiO_2_ photocatalyst using the same overall mass (i.e., total mass of TiO_2_-carbon composite photocatalyst = mass of pure TiO_2_ photocatalyst, in the case of our Figure 6, 32.5 mg of catalyst). In contrast, some other authors prefer to compare the solids using the same mass of TiO_2_. Following this latter approach, the activity of the solids is expressed in Figure 7 per gram of TiO_2._ This figure shows how the most active catalysts are those that present a combination of the SG1 method and the use of functionalized mesoporous carbon (MC-H) as a support for TiO_2_. This combination leads to an optimal dispersion of TiO_2_ on the surface of the carbonaceous support and as a consequence, maximizes the absorption capacity of a light photon and the formation of the e^−^/h^+^ pair, whose charge separation is favored by the presence of MC-H in intimate contact with TiO_2_. On the other hand, catalysts synthesized on unfunctionalized MC present an intermediate activity that can be associated with a lower hydrophilicity of the semiconductor [34,35,39]. Furthermore, the catalyst synthesized by SG1 is more active than the equivalent synthesized by the SG2 method, with a much more aggressive hydrolysis of titanium isopropoxide as a consequence of the addition of water during synthesis. This difference in reactivity between the SG1 and SG2 methods is therefore associated with the effect of the better TiO_2_ dispersion achieved by SG1 as a consequence of the much slower hydrolysis of the titanium precursor.

Finally, the catalyst synthesized by physical mixing only slightly improves the production capacity of hydrogen per gram of TiO_2_ of P25, as a consequence of a null dispersion of TiO_2_ that is simply deposited on the MC and with less efficient contact between both components.

There are several examples in the literature of the use of TiO_2_/C composites for the production of hydrogen [68,69,70,71]. However, it is difficult to compare the results, since there are different factors to consider, such as the alcohol used as a sacrificial agent (typically MeOH) and its concentration, the TiO_2_ content, the type and intensity of radiation used, etc. [72,73,74]. For this reason, it seems more convenient to compare the results obtained under each specific reaction condition, taking a standard catalyst, such as P25, as the reference [68]. In this sense, hydrogen production with the solid SG1(25%)TiO_2_/MC-H is about 11 times greater than with P25, expressed per gram of catalyst and per hour. This improvement factor is higher than that found by Hakamizadeh et al. [68]. Likewise, the low content of TiO_2_ in our system (ca. 10% wt.) is noteworthy. Therefore, if hydrogen production is expressed per gram of TiO_2_ and per hour, the improvement factor compared to P25 is 110 (Figure 7). If the physical mixture (PM(40%)TiO_2_/MC) is taken as the reference, there is a 39-fold increase, which evidences the importance of an appropriate control of the synthetic method for photocatalytic activity.

## 4. Conclusions

Different TiO_2_-carbon composites were synthesized in this study, starting from untreated or activated (acid-treated) mesoporous carbon. Titania was incorporated as a physical mixture (PM solid) or through the sol-gel process, using titanium isopropoxide as the Ti precursor (with or without the addition of water named SG2 and SG1 series, respectively). The activation of mesoporous carbon with HNO_3_/H_2_SO_4_ resulted in an increase in the surface acidity (especially strong carboxylic groups) and mean pore diameter, whereas the BET surface area hardly changed. On the contrary, the subsequent incorporation of titania resulted in a decrease of the BET surface area, probably due to the partial clogging of mesopores by titania. Titania incorporation through the SG1 method seems to be limited by the active sites on the carbon surface, whereas the SG2 method ensures quantitative incorporation though less intimate carbon-titania contact. SEM, IR, and Raman techniques evidenced a very homogeneous distribution of small titania crystals on mesoporous carbon for SG1 solids, whereas bigger and more heterogeneously-distributed titania particles were obtained for SG2 and PM series. In all cases, the presence of carbon resulted in a shift of light absorption to the visible range (gap energies of 2.25–2.37 eV). The solids were tested for hydrogen production through glycerol photoreforming, with the highest hydrogen production values, expressed per gram of titania, being achieved with the solids obtained on activated mesoporous carbon and through the SG1 method. Therefore, the most active solid exhibited an activity more than 100 times higher than P25. All in all, these results evidence the important role of the titania incorporation method in TiO_2_/C composites, with the development of highly-dispersed, homogeneously distributed titania particles being crucial.

## Figures and Tables

**Table 1:** Acidic properties (Boehm titration), as well as the pH at the point of zero charge (PZC), of non-functionalized mesoporous carbon (MC) and functionalized mesoporous carbon (MC-H) solids.

Mesoporous Carbon	Washing pH	Carboxylic Groups (mmol/g) *	Lactonic Groups (mmol/g) *	Phenolic Groups (mmol/g) *	Total Acidity (mmol/g)	pH_pzc_
MC-H	1.50	7.02 (39.5%)	4.33 (24.4%)	6.41 (36.1%)	17.76	2.80
MC-H	2.00	6.96 (42.5%)	4.03 (24.7%)	5.37 (32.8%)	16.36	3.45
MC-H	3.00	6.02 (42.4%)	3.32 (23.3%)	4.88 (34.3%)	14.22	3.66
MC	-	4.87 (36.4%)	3.11 (23.3%)	5.38 (40.3%)	13.35	6.80

* The number in brackets corresponds to the relative percentage of the considered acidic group.

**Table 2:** Titanium dioxide (TiO_2_) and sulfur content, Brunauer–Emmett–Teller (BET) surface, and band gap of the different solids.

Catalyst	TiO_2_ (wt.%)	S (wt.%)	S_BET_ (m^2^·g^−1^)	Band Gap (eV)
SG1(10%)TiO_2_/MC-H	6.63	1.63	147	2.37
SG1(25%)TiO_2_/MC-H	9.86	0.63	192	2.25
SG1(40%)TiO_2_/MC-H	10.47	1.27	207	2.35
SG1(40%)TiO_2_/MC	16.47	0.47	188	2.31
SG2(40%)TiO_2_/MC	43.56	0.34	176	2.29
PM(40%)TiO_2_/MC	40.98	0.85	153	2.34
MC	-	0.96	221	-
MC-H	-	1.24	220	-

**Table 3:** X-ray photoelectron spectroscopy (XPS) results for SG1(40%)TiO_2_/MC-H and SG1(40%)TiO_2_/MC.

Signal	Assignment	SG1(40%)TiO_2_/MC-H			SG1(40%)TiO_2_/MC		
		Binding Energy	% Area	Total Atomic %	Binding Energy	% Area	Total Atomic %
C1s	C (sp^2^)	284.6	62.4	48.45	284.6	65.9	60.70
	C (sp^3^)	285.5	12.7		285.8	11.4	
	–OH	286.6	8.0		286.9	6.4	
	C=O	287.7	5.6		288.0	4.0	
	–COOH	288.7	3.2		289.1	2.6	
	π-π*	290.5	8.1		291.1	9.7	
Ti2p3/2	Ti^4+^	459.0	100	11.01	459.0	100	10.84
O1s	Ti–O–Ti, S–O	530.7	44.3	39.22	530.4	61.9	27.88
	Ti–OH, C=O	532.1	34.5		532.1	23.4	
	C–O, C–OH, (C–O–C)	533.4	18.6		533.4	11.5	
	O–C=O, SOx, Chemisorbed oxygen and/or water	535.3	2.6		535.9	3.2	
S2p3/2	S_8_, C–S, aromatic sulfide	164.6	6.8	1.32	164.1	43.7	0.57
	–SO_3_	169.5	93.2		169.4	56.3	

**Table 4:** Textural properties of the different catalysts used in the present study.

Sample	Pore Diameter (nm)	V_total_ (mL·g^−1^)	V_micro_ (mL· g^−1^)	V_meso_ (mL·g^−1^)	Micropores (%)	Mesopores (%)
SG1(10%)TiO_2_/MC-H	13.2	0.44	0.01	0.43	2	98
SG1(25%)TiO_2_/MC-H	11.0	0.46	0.02	0.44	4	96
SG1(40%)TiO_2_/MC-H	23.5	0.35	0.02	0.33	6	94
SG1(40%)TiO_2_/MC	16.7	0.36	0.02	0.34	5	95
SG2(40%)TiO_2_/MC	14.6	0.65	0.01	0.64	2	98
PM(40%)TiO_2_/MC	26.4	0.37	0.01	0.36	4	96
MC	14.2	0.79	0.03	0.76	3	97
MC-H	20.8	1.14	0.02	1.12	2	98

## Supplementary Materials

The following are available online at https://www.mdpi.com/1996-1944/13/17/3800/s1: Figure S1: Tauc Plot for the calculation of the band gap energies of the different TiO_2_-containing catalysts, Figure S2: Accumulated hydrogen production in the most active system, SG1(25%)TiO_2_/MC-H, over 20 h.
