# Supplementary material for: Hydrogen Production through Glycerol Photoreforming on TiO2/Mesoporous Carbon: Influence of the Synthetic Method
Source: Materials (Basel). 2020 Aug 28;13(17):3800. doi: 10.3390/ma13173800 (PMC7504067; doi:10.3390/ma13173800)
Supplement: Supplementary file 1 [file materials-13-03800-s001.pdf]

materials

Supplementary Materials

Hydrogen Production Through Glycerol Photoreforming on TiO_2/Mesoporous Carbons: Influence of the Synthetic Method

Juan Carlos Escamilla, Jesús Hidalgo-Carrillo, Juan Martín-Gómez, Rafael C. Estévez-Toledano, Vicente Montes, Daniel Cosano, Francisco J. Urbano and Alberto Marinas

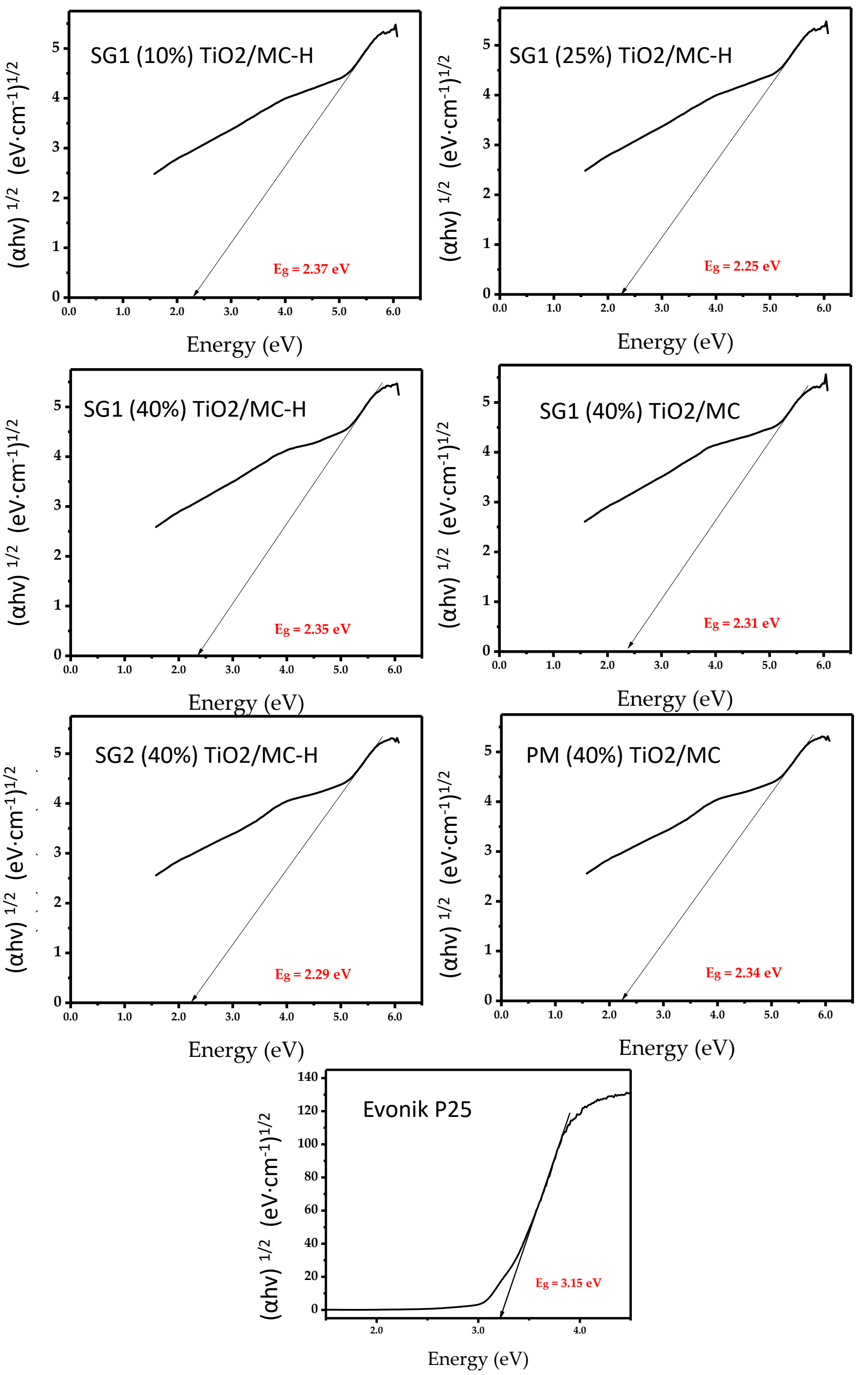

Figure S1. Tauc Plot for the calculation of the band gap energies of the different TiO_2-containing catalysts.

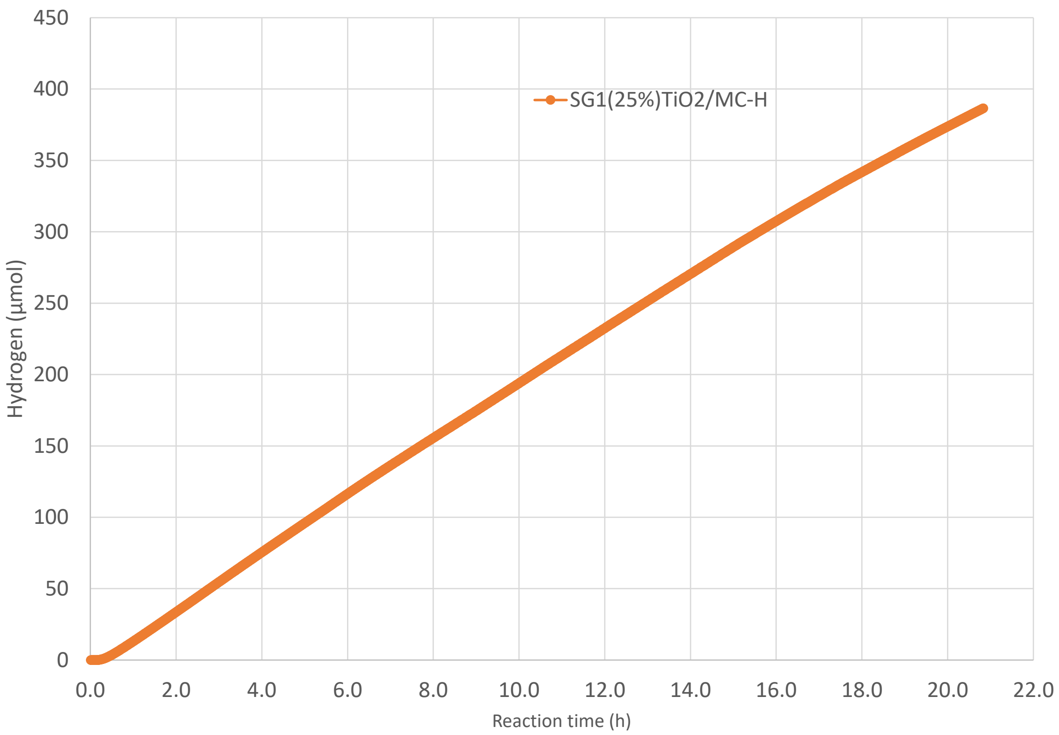

Figure S2. Accumulated hydrogen production on the most active system, SG1(25%)TiO_2/MC-H, during over 20 h.

© 2020 by the authors. Submitted for possible open access publication under the terms and conditions of the Creative Commons Attribution (CC BY) license (http://creativecommons.org/licenses/by/4.0/).
